# Supplementary material for: Cross-reactive antibody response between SARS-CoV-2 and SARS-CoV infections
Source: bioRxiv. 2020 Mar 17:2020.03.15.993097. Preprint. [Version 1] doi: 10.1101/2020.03.15.993097 (PMC7239046; doi:10.1101/2020.03.15.993097)
Supplement: Supplement 1 [file media-1.pdf]

Supplementary Table 1. Patient information, ELISA data, and neutralization data.

				ELISA (OD_{450} value at 1:100 dilution)				MN titer	
**Patient ID**	**Gender**	**Age**	**day post-symptom onset**	**SARS-CoV-2 spike**	**SARS-CoV spike**	**SARS-CoV-2 RBD**	**SARS-CoV RBD**	**SARS-CoV-2**	**SARS-CoV**
#1	M	56	6	0.30	0.21	0.03	0.01	<1:10	<1:10
#2	F	62	4	0.32	0.23	0.07	0.03	<1:10	<1:10
#3	F	62	22	1.97	0.66	1.46	0.12	1:160	<1:10
#4	M	63	22	1.95	0.98	1.56	0.58	1:80	<1:10
#5	M	47	8	0.52	0.40	0.20	0.03	1:80	<1:10
#6	M	64	19	1.81	0.54	1.40	0.50	1:320	<1:10
#7	F	73	18	1.60	0.71	0.90	0.17	1:80	<1:10
#8	M	72	18	1.94	1.19	1.21	0.14	1:80	<1:10
#9	F	37	2	-0.25	-0.10	-0.05	-0.39	<1:10	<1:10
#10	F	72	10	1.44	0.41	1.04	-0.03	1:40	<1:10
#11	M	60	22	2.53	1.38	2.24	1.15	1:160	<1:10
#12	M	56	13	1.57	0.64	1.37	0.00	1:320	<1:10
#13	F	55	11	0.03	-0.09	0.05	0.08	<1:10	<1:10
#14	F	63	19	2.44	1.12	2.06	1.42	1:640	<1:10
#15	M	37	14	1.85	1.60	1.43	0.59	1:320	1:10

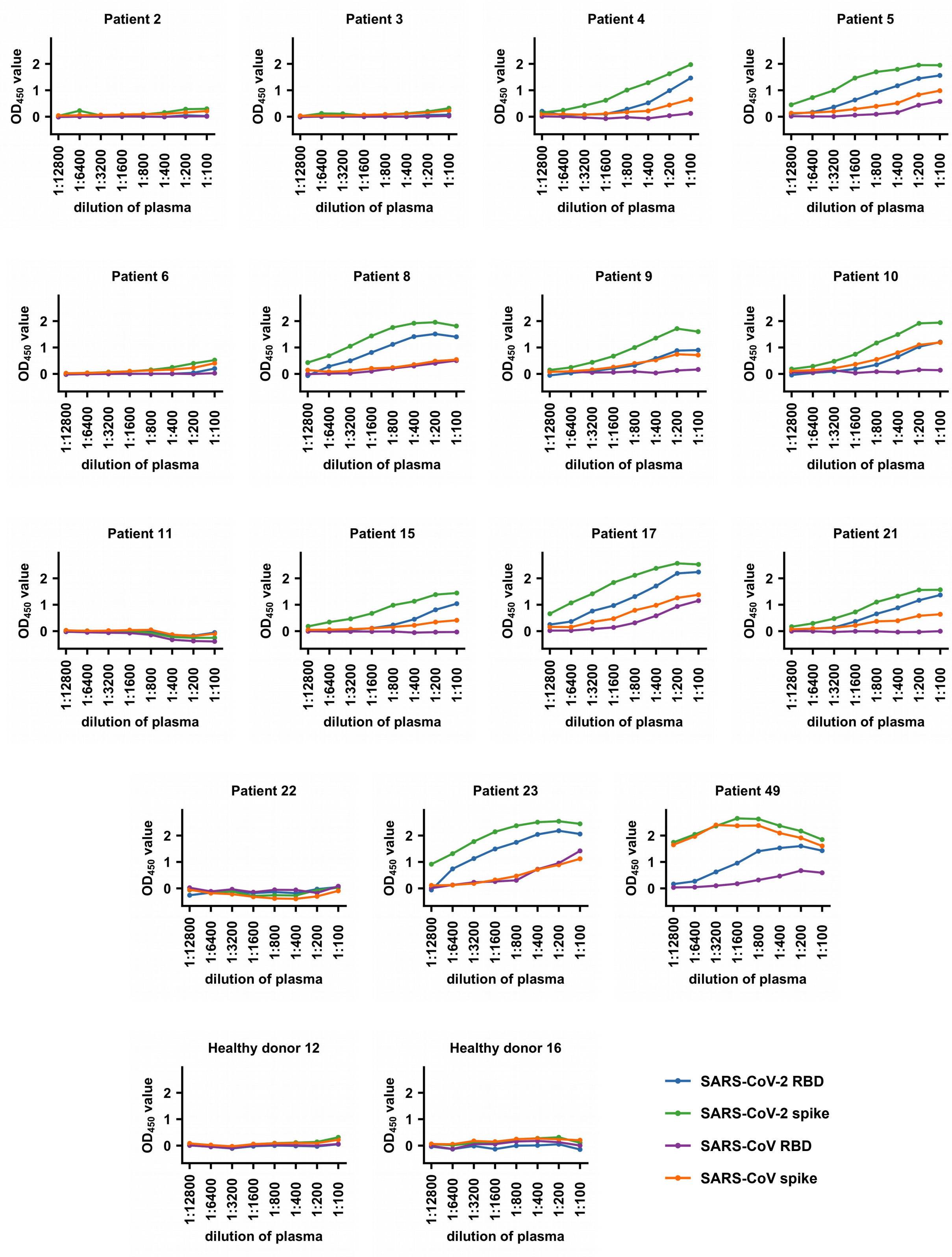

Supplementary Figure 1. ELISA of patient samples at different dilutions. Binding of different dilutions of plasma samples from patients and healthy donors to spike and RBD from SARS-CoV-2 and SARS-CoV was measured by ELISA. The mean OD_{450} values calculated after testing each plasma sample in duplicate are shown.

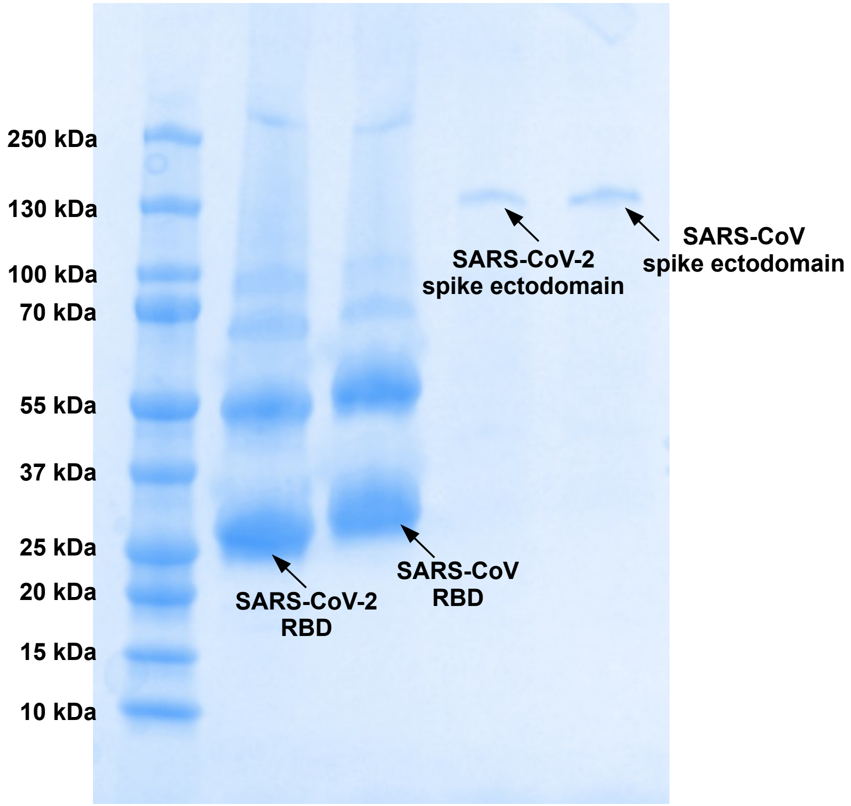

Supplementary Figure 2. Protein expression. Proteins purified by Ni-NTA beads from insect cell expression supernatant were analyzed by coomassie staining. The proteins were further purified by size exclusion chromatography before used.
